# Effects of the vaccination against SARS-CoV-2 on infections and on hospitalizations in European countries

**DOI:** 10.1007/s43546-023-00445-0

**Published:** 2023-02-10

**Authors:** Alfred Greiner, Benjamin Owusu

**Affiliations:** Department of Business Administration and Economics, Bielefeld University, Bielefeld, Germany

**Keywords:** COVID-19, Vaccination, Pandemic, Externalities, C10, D62, I10

## Abstract

Since December 2020 vaccines against the SARS-CoV-2 virus have been available. However, little is known regarding their effects on infections and on hospitalizations. To gain insight into this topic we empirically analyze the effects of the vaccinations against SARS-CoV-2 for European countries beginning 2021 to February 2022 with weekly data. We perform panel fixed effects estimations, GMM estimations and nonlinear penalized spline estimations. We find a statistically significant and positive relationship between the share of infections with the SARS-CoV-2 virus and the share of vaccinated people in nine estimations while one estimation output was insignificant. Regarding hospitalizations, six out of ten estimations yielded a statistically insignificant relationship, and three estimation results were weakly statistically significant with a negative coefficient and one indicated a statistically significant negative relation. Hence, there is empirical evidence for a positive relationship between infections and the share of vaccinated people whereas we find weak empirical evidence for a negative relation between vaccinations and hospitalizations. The implication of our analysis is that vaccinations alone cannot end the pandemic. Rather developing effective medicines should be seen as an additional measure.

**Supplementary Information:**

The online version contains supplementary material available at 10.1007/s43546-023-00445-0.

## Introduction

The COVID-19 pandemic started in Wuhan/China in 2019 and is still going on. In the European Union (EU) vaccinations against the SARS-CoV-2 virus have begun in December 2020. Until March 2022, the 5 vaccines Comirnaty, Nuvaxovid, COVID-19 Vaccine Janssen, Spikevax and Vaxzevria have received authorization in the EU (see European Medicines Agency 2022).

Under ideal circumstances, vaccinations do not only protect the vaccinated person from getting ill, but, go along with a positive externality, meaning that other people are protected since the vaccinated persons cannot get infected and, therefore, do not transmit the virus. As regards the vaccination against COVID-19, however, it is questionable whether it is associated with such positive external effects since it has turned out that those who have received it can nevertheless get infected and transmit the virus (see e.g. Acharya et al. 2021; Kampf 2021; Singanayagam et al. 2021; Subramanian and Kumar 2021).

A reason for that observation may be that the effectiveness of the vaccinations declines progressively over time as shown by Nordström et al. (2021). Those authors show that, in the case of Vaxzevria produced by AstraZeneca, the probability of getting infected is higher for vaccinated persons than for non-vaccinated ones after 180 days. The reason for that is unclear: It may be due to the so-called ’antibody-dependent enhancement’ or to the ’vaccine-associated hypersensitivity’ effects or due to the fact that vaccinated behave less carefully and stick to a lesser extent to the hygiene measures. Another reason may simply be statistical uncertainty since a part of the 95% confidence interval still lies in the positive range.

With this paper we want to contribute to the analysis of the question whether the vaccinations against COVID-19 imply a positive externality and, thus, can contribute to end the pandemic. To do so, we estimate the relationship between the 7 day infection rate, the number of fully vaccinated people and the number of persons having received a booster injection, respectively, where we control for the effects of other variables. In addition, we estimate the effect of the vaccinations on the hospitalization rate to find out whether the vaccinations reduce that rate because hospitalization is an important factor that helps to assess the severity of a pandemic. To see how reliable the estimated coefficients are, we resort to three different estimation methods: panel fixed effects estimation, generalized method of moments (GMM) estimation and penalized spline estimation.

The rest of the paper is organized as follows. In the next section, we present some literature dealing with the effects of COVID-19 and we point out how our study helps to fill a gap in the literature. Section 3 gives the theoretical background where we point out which factors are expected to influence the infection rate and the rate of hospitalizations and we comment on the data used for the research. Section 4 presents the results of our estimations and the interpretation and section 5, finally, gives a brief summary and concludes. In the Appendix 6 we report some additional technical relationships.

## Literature on the effects of COVID-19

Meanwhile, there exists an exhaustive literature dealing with the effects of COVID-19. Hence, it is virtually impossible to give a complete survey of the existing studies. Therefore, we confine our presentation to some papers that provide insight into the topic.

On the one hand, there are studies analyzing COVID-19 as regards its economic effects. For example, Agrawal and Bütikofer (2022) illustrate how COVID-19 has affected public economics with a special focus on the labor market and the effects of technical change. In addition, they point out the role of fiscal federalism in this context and they discuss various policy responses to the pandemic. A different perspective has been taken by Adebayo et al. (2022) who analyze the effects of CO$_2$ emissions in the United Kingdom during the COVID-19 pandemic with a special emphasis on the role played by renewable energy. They find, among other things, that emissions have declined with a higher number of COVID-19 cases due to lockdown measures. Bo (2021) studies the role of subnational autonomy with respect to the fatal consequences of the pandemic, while controlling for other relevant factors. He finds that countries with higher degrees of subnational autonomy are experiencing higher deaths.

On the other hand, studies have been written analyzing which factors determine the evolution of the COVID-19 pandemic over time. Doroshenko (2021) presents evidence that nonpharmaceutical interventions (NPIs), such as isolation and quarantine, limiting social interactions, and enhanced respiratory hygiene, minimìze the impact of the pandemic by reducing the effective transmission rate. Further, based on mathematical models from the literature he concludes that vaccinations reduce the number of deaths. Another study analyzing the effects of NPIs is provided by Mader and Ruettenauer (2022). They find that many implemented NPIs may not have reduced COVID-19 deaths directly, but, NPIs can have reduced the number of deaths by preventing exponential growth. As regards vaccinations they find that those were effective in reducing the number of COVID-19 deaths. Hirotsu et al. (2022) analyze the effects of vaccinations against SARS-CoV-2 with respect to the virus load of persons infected with the Omicron SARS-CoV-2 virus. They found that fully vaccinated people (three doses) had viral loads similar to unvaccinated patients or who received only two doses. Hence, they conclude that this can explain breakthrough infection with the Omicron variant. A similar result has been obtained by Acharya et al. (2021) who studied two demographically distinct populations in California during a 2 month period. They did not find a significant difference in the virus load between vaccinated and unvaccinated people infected with the SARS-CoV-2 Delta variant. A similar result was obtained by Singanayagam et al. (2021). They detected that it is true that full vaccination reduces the risk of an infection with the Delta variant. But, nevertheless, fully vaccinated individuals with breakthrough infections have a peak viral load similar to unvaccinated cases and can efficiently transmit the infection. Subramanian and Kumar (2021) do not find a negative relationship between the percentage of population fully vaccinated and new COVID-19 cases, but rather the opposite. They draw a trend line through the observed data that suggests a slightly positive relation such that countries with higher a proportion of population fully vaccinated have higher COVID-19 cases per 1 million people. Rzymski et al. (2022) examined whether vaccine coverage in the European Economic Community countries are associated with COVID-19 related burden (in terms of infection rates, hospitalizations, admission to intensive care units and deaths), where they considered the wave of the Omicron variant from January to April 2022. They concluded that the vaccination (including boosters) is related to a decreased COVID-19 burden during the first 2 months of 2022. However, with the exception of intensive care unit admissions, the negative correlation between vaccinations and the other related COVID burden (hospitalizations and death due to COVID-19) diminished in the subsequent months (March and April). Additionally, their results show the absence of correlation between the infection rate and the vaccine coverage in any of the months studied.

Looking at the empirical literature dealing with the SARS-CoV-2 pandemic and the ways how to overcome it, one realizes that there does not exist a fully-fledged econometric study analyzing the effects of vaccinations on the infection rate and on the rate of hospitalization, to our knowledge. The study by Rzymski et al. (2022) is similar to our study in terms of research objectives. However, they only studied the correlation between the variables (within a shorter time frame) without estimating any econometric specification. Secondly, they focus specifically on the SARS-CoV-2 Omicron variant. We analyze the relationship between the rate of vaccinations and the rate of infection and the hospitalization rate, respectively, where we control for the effect of other variables that can affect infections and hospitalizations. Thus, we intend to contribute to the research on the effectiveness of the vaccination against SARS-CoV-2.

## Theoretical background and data

In this section, we briefly look at the theoretical relationship between vaccinations, the control variables and the infection rate, on the one hand, and the rate of hospitalization, on the other hand. Furthermore, we give a brief description of the data used for the research.

Firstly, we are interested in the effect of vaccinations against COVID-19 on the rate of infections. To do so we perform a panel data analysis where we regress the number of infections over the last 7 days (given as a rolling 7 day average) per 100 people in the total population, which we call the infection rate or briefly the infections.^1^ Our explanatory variable of interest is the total number of people who received all doses prescribed by the vaccination protocol per 100 people in the total population, which we refer to as vaccinations. In a separate regression, we use as the explanatory variable the total number of COVID-19 vaccination booster doses administered per 100 people in the total population we term as boosters. Since the reports of new infections are not always the same over the week, for example over the weekend there are less reported numbers, we use weekly data with one observation obtained as the aggregate of the 7 daily observations.

As a control variable we include lockdown measures that governments have taken to reduce the number of contacts between people and, consequently, the number of infections. As regards the efficacy of lockdown measures, Alfano and Ercolano (2020) have found in a cross-country panel analysis that lockdown measures are effective 10 days after the implementation of the policy. Medeiros de Figueiredo et al. (202) studied the effects of lockdown measures with respect to the incidence and to the mortality rate in the two Chinese provinces Hubei and Guangdong and found a daily relative risk reduction with a 7 day time-lag in Guangdong and with a 17 day time-lag in Hubei. This demonstrates that the measures work with a certain time lag which we take into account in our model by including the policy from the previous period $t-1$, i.e. from the previous week, we denote as lagged government policy. Lockdowns are measured by the government response stringency index: a composite measure based on 9 response indicators such as school closures, workplace closures, and travel bans rescaled to a value from 0 to 100 (100 = strictest response) taken from Ritchi et al. (2020). We have constructed the variable for one week, i.e. one observation, by computing the arithmetic mean of the 7 daily observations.

Further, we allow for the seasons winter, spring, summer and autumn. We do so because infection rates are typically lower in warmer periods than in colder ones (see for example Nichols et al. 2021). Finally, the number of new tests per 1000 people in the total population is used as an additional explanatory variable. The reason for that is that the infection rate rises if more tests are performed, simply because more infections are detected. Therefore, we control for the number of tests in our regressions, too, called testing.

As regards the rate of hospitalization,^2^ new hospitalizations per million, our main variables of interest are again the share of vaccinated people and the share of people having received a booster injection. As control variables we again include the number of tests and the infection rate (infections) which is assumed to have a positive effect on the hospitalization rate. In addition, we control for the seasons and for governmental lockdown measures. We should like to point out that the share of older people is implicitly taken into account in the fixed effects model since that variable is treated as an individual fixed effect. Further, we stress that the number of hospitalizations is relative to the total population and not relative to infected persons only. We use that variable because we are interested in the societal effects and not in the medical effects on the individual level.

## Empirical analysis and interpretation

This section presents the empirical methodology, the estimation and the discussion of our results. Our empirical estimation follows a panel data analysis of countries that are considered geographically as a part of Europe. We attempt to answer the following research questions with empirical data to understand the role of vaccinations and boosters in ending the COVID-19 pandemic:

i) How have the vaccinations impacted COVID-19 related cases?
ii) Have the vaccinations reduced the COVID-19 related hospitalization?

Hence we will estimate two main general regression models given by

1 $$\begin{aligned} I_{i,t} = \alpha _{i} +\beta V_{i,t} + \phi Z_{i,t}^{T}+ \sum _{h=1}^{3}\gamma _{h} D_{h} + \epsilon _{i,t} \end{aligned}$$

where *i* represents the individual countries in the panel, *t* is the time dimension and *I* represents our dependent variable of interest. We estimate two regression models; in the first model *I* is the number of COVID-19 related infections whereas in the second model it represents the number of hospitalizations due to COVID-19. We do this to answer the above-mentioned research questions. We resort to a one-way error component model such that $\alpha$ represents the individual fixed effects. We augment the model with seasonal dummies to capture the effects of the seasons on the COVID-19 pandemic.

*V* is the number of people fully vaccinated per hundred of the population whilst $\beta$ measures the impact of vaccinations on infections. In a separate regression, we also consider the number of boosters per hundred of the population as our main regressor of interest to understand its impact on COVID-19 infections. The vector *Z* represents all control variables in the model, $\phi$ is the coefficient measuring its impact on the infections and $\epsilon$ represents the error term. *D* represents the seasonal dummies namely: winter, spring and summer. It should be noted that we omit the dummy for autumn from our estimation to avoid multicollinearity between the intercept and one of the dummy variables, a fact also known as the dummy variable trap (see Gujarati 1970). All variables are assumed to be drawn from an identically and independently distributed (iid) process so that the error or residuals follow an iid process with a zero mean and a constant variance. We also postulate that each variable carries unique information and hence the absence of multicollinearity.

Firstly, we assume all variables on the right hand side (RHS) of equation (1) enter the model linearly, hence we posit a linear relationship between infections and hospitalizations, respectively, and all the regressors. Later on, in the subsequent subsection, we shall relax the above linearity assumption by estimating a semi-parametric regression where we resort to penalized spline estimations.

Since the regressors exist in different units and, therefore, have different scales, the interpretation of the regression coefficients is sensitive to the scale of input variables (Gelman 2008). Hence, regressors with different input scales in the same model have the tendency of producing biased regression estimates. It is prudent to standardize and put all regressors on the same scale (James et al. 2013). We therefore standardized the regressors by subtracting data points from their respective means and dividing by the standard deviation. The transformed variables are zero mean centered with unit variance.^3^

To begin our analysis, we compute the correlations that exist between all the variables. Table 9 in the Appendix reveals that the chance of multicollinearity is very small since none of the correlation coefficients exceeds 70%. This fulfills our assumption that each variable carries unique information and, therefore, the absence of multicollinearity is assured. A positive relationship is observed between infections and vaccinations and boosters, respectively. The same holds for the relation between hospitalizations and boosters, while we observe a negative correlation between hospitalizations and vaccinations. Further, a pairwise scatter plot (figure 1 in the appendix) does not explicitly reveal the directions of the relationship between the variables. To obtain deeper insight into the relations between those variables we next estimate several regression models.

### Linear specifications

We begin by testing all variables for stationarity to avoid making inferences from spurious regression. We employ two panel stationarity tests, the Im, Pesaram and Shi (IPS) test and the Levin, Lin and Chu test (LLC) which are widely used in panel data studies. Both tests overwhelmingly reject the null hypothesis of a unit root in favour of the alternative hypothesis of stationarity. Table 6 in the Appendix provides a report of both tests. All variables are stationary irrespective of whether we consider a model with a constant or slope.

In the first linear specification, we estimate (1) with the help of a panel fixed effects model with individual effects. In order to employ the panel fixed effects model, we also assume that the slope coefficient of (1) can be pooled across all the cross-sections. This implies a homogeneous slope coefficient which can be justified by the fact that we consider countries in the same European region with similar characteristics.

Our justification for using panel fixed effects stems from the fact that we consider a sample of European countries with individual characteristics, which can be cultural effects for instance or country-wise political effects. We believe these individual country characteristics could have an impact on the response variable. Hence, using the fixed effect model enables us to control for these individual country-wise characteristics.

Secondly, we provide an econometric justification for using fixed effects over the alternative random effects using the so-called Hausman test according to Hausman’s 1978 specification test. We test the null hypothesis that the individual unique errors are not correlated with the regressors against an alternative hypothesis that they are indeed correlated. Results of the random effects model and the Hausman test can be found in Table 7 and Table 8 in the appendix respectively. From Table 8, we can observe a statistically significant p-value which indicates the rejection of the null hypotheses ($H_{0}$: individual errors are not correlated with the regressors). Hence, the fixed effect model is suitable as compared to a random effect model and hence a justification of our chosen model. Additionally, to further ensure the model is robust against multicollinearity, we estimate the variance inflation factor (vif) for each of the regressors in our fixed effect model (Table 9 in the appendix). The vif quantifies how much the variance (or the standard error) is inflated due to the presence of multicollinearity. It can be observed that the vif is less than 2 units, indicating the absence of high multicollinearity.

Given the presence of a lagged regressor, one could argue that the model is dynamic which implies a possible correlation between the lags of the regressors and the error term. Secondly, in the presence of potential endogeneity arising as a result of correlation between any of the regressors and the error term, static estimators such as the fixed effects estimator could provide biased estimates. Hence we consider an alternative estimator suitable for dynamic models in the like of the generalized method of moments (GMM) where we instrument the model using lags of the regressors to circumvent a possible endogeneity bias. Exploiting moments conditions via GMM has proven to deal with the issue of endogeneity emanating from dynamic models more efficiently and has been largely used in most of the empirical econometrics literature. We specifically resort to the system GMM propounded by Blundell and Bond (1998). Table 1 gives the results of the estimations. Fixed effects standard errors are heteroskedastic and autocorrelation consistent (HAC) according to the Newey–West method (Newey and West 1987). Similarly, GMM standard errors are robust against heteroskedasticity and autocorrelation. The sample period comprises 60 weeks from the beginning of 2021 to February 2022 made up of 32 European countries for infections and fully vaccinated people and for boosters we have 27 countries for 26 weeks from September 2021 to February 2022.^4^

**Table 1:** Estimation results - Parametric/Linear Specification

	*Response variable: Infections*				
	*Panel Fixed Effects*		*Panel GMM*		
*Variables*	1	2	3	4	5
*Vaccination*	0.133***		0.112**	0.144	
	(0.037)		(0.045)	(0.108)	
*Testing*	0.445***	0.411***	0.461***	0.482***	0.383***
	(0.048)	(0.054)	(0.070)	(0.101)	(0.091)
*Lagged Govt Policy*	$-$0.130***	$-$0.281***	$-$0.191***	$-$0.230***	$-$0.251***
	(0.039)	(0.059)	(0.341)	(0.040)	(0.083)
*Spring*	0.192***		0.018	0.078	
	(0.071)		(0.046)	(0.096)	
*Summer*	$-$0.284***		$-$0.504***	$-$0.489***	
	(0.050)		(0.048)	(0.096)	
*Winter*	0.649***	0.239**	0.474***	0.470***	0.118
	(0.077)	(0.117)	(0.045)	(0.077)	(0.196)
*Booster*		0.471***			0.496***
		(0.061)			(0.155)
*Adj* $R^{2}$	0.44	0.57			
*Observ*	1,920	702	3,744	3,744	1,225
*Sargan Test*			32(0.99)	31.5(0.99)	24.21(0.99)
*Autoc test (1)*			1.90(0.06)	$-$1.77(0.08)	$-$0.83(0.41)
*Autoc test (2)*			$-$0.54(0.590 )	$-$0.78(0.44)	$-$2.11(0.03)
*Wald test of coefficients*			2097.2(0.000)	772.3(0.000)	536.4(0.000)

HAC standard errors are indicated in parenthesis. *, ** and *** indicates statistical significance at 10%, 5% and 1%, respectively

Model 1 – Fixed effects with individual effects. Seasonal dummies included as control

Model 2 – Fixed effects with individual effects (using boosters as regressors)

Model 3 - Panel system GMM based on Blundell and Bond estimator (one step process)

Model 4 - Panel system GMM based on Blundell and Bond estimator (two step process)

Model 5 - Panel system GMM based on Blundell and Bond estimator (using boosters as regressors. Two step process)

It can be seen that, except for model 4 in table 1, both the share of fully vaccinated and the share of boosters are statistically significant and positively correlated with the infections. This implies that neither double vaccinations nor boosters reduce the rate of infections. Rather, it seems that the inverse holds: the more people have received the vaccination the higher the infection rate. This result is in line with the outcome obtained by Subramanian and Kumar (2021) who find a slightly positive trend line showing the relation between the number of vaccinated people and the infections.

Our conjecture for that outcome is that the vaccinations cannot prevent infections and the spread of the SARS-CoV-2 virus in the community, as shown by Acharya et al. (2022) and by Singanayagam et al. (2021). In addition, vaccinated people often do not need a COVID-19 test certificate to participate in social life, thus, raising the number of infections. Further, it might be that vaccinated people behave less carefully since the vaccinations give them a sense of security that, however, is not provided by the COVID-19 vaccines. We underline that this is a conjecture and that our estimation results allow statements with respect to the correlation between variables, but, not necessarily with respect to causality.

Another fact that can contribute to this result is the following. It turned out that vaccinated persons and previously infected individuals can be just as likely to transmit the virus as unvaccinated and not previously infected ones. Hence, infection-induced immunity is associated with protection against infection, but, not necessarily with the absence of the transmission of the virus (see Frutos et al. 2022).

As regards the other variables we obtain the expected sign for the coefficients. The number of tests is positively correlated with the number of infections, i.e. the more tests are performed the more infections are detected, and lockdown measures seem to reduce the infection rate in the consecutive time period, thus supporting the result in Doroshenko (2021). The latter effect is highly statistically significant and holds for all estimated models. Finally, the rate of infections declines in summer and rises in winter, as was to be expected since this has already been shown for England and Wales in the paper by Nichols et al. (2021).

**Table 2:** Estimation results - Parametric/Linear Specification

	*Response variable: Hospitalizations*				
	*Panel Fixed Effects*		*Panel GMM*		
*Variables*	1	2	3	4	5
*Vaccination*	$-$0.134*		$-$0.137	0.263	
	(0.073)		(0.085)	(0.299)	
*Testing*	0.007	0.205***	0.001	0.538*	0.174***
	(0.073)	(0.053)	(0.087)	(0.289)	(0.087)
*Lagged Govt Policy*	0.017	0.0005	0.164***	0.168	0.072
	(0.078)	(0.046)	(0.058)	(0.136)	(0.044)
*Infections*	0.526***	0.428***	0.594***	$-$0.183	0.455***
	(0.080)	(0.057)	(0.089)	(0.314)	(0.062)
*Spring*	0.533**		0.209	$-$0.143	
	(0.208)		(0.146)	(0.411)	
*Summer*	$-$0.397***		$-$0.552***	$-$1.207***	
	(0.104)		(0.096)	(0.314)	
*Winter*	0.626***	0.665***	0.259***	0.119	0.198
	(0.143)	(0.129)	(0.161)	(0.446)	(0.191)
*Booster*		$-$0.131*			$-$0.062
		(0.069)			(0.141)
*Adj* $R^{2}$	0.61	0.69			
*Observ*	480	208	936	936	392
*Sargan Test*			8.0(0.99)	1.60(0.99)	8.0(0.99)
*Autoc test (1)*			$-$0.11(0.91)	2.57(0.010)	$-$1.11(0.27)
*Autoc test (2)*			$-$0.4(0.69 )	1.45(0.15)	$-$1.32(0.19)
*Wald test of coefficients*			3905(0.000)	453.6(0.000)	338.7(0.000)

HAC standard errors are indicated in parenthesis. *, ** and *** indicates statistical significance at 10%, 5% and 1%, respectively

Model 1 – Fixed effects with individual effects. Seasonal dummies included as control

Model 2 – Fixed effects with individual effects (using boosters as regressors)

Model 3 - Panel system GMM based on Blundell and Bond estimator (one step process)

Model 4 - Panel system GMM based on Blundell and Bond estimator (two step process)

Model 5 - Panel system GMM based on Blundell and Bond estimator (using boosters as regressors for two step procedure))

In table 2 we present the estimation outcome with the rate of hospitalizations as the dependent variable.^5^ It can be seen that we obtain a negative relationship between hospitalizations and vaccinations and boosters in the fixed effects model. However, the statistical significance is low. But, the GMM model does not yield a statistically significant relationship between those variables. As regards the control variables we see that the rate of infections exerts a statistically significant and positive effect on hospitalizations, except for model 4, and the seasons summer and winter have again a negative and positive effect, respectively. In model 3 lockdown measures, i.e. Lagged Govt Policy, is statistically significant and positive which could be explained by the fact that with a tense COVID-19 situation both hospitalizations and lockdown measures rise.

In table 6 in the Appendix we report the estimation result with the non-standardized variables, where the statistically significant and positive relation between infections and vaccinations is confirmed while the relationship between hospitalizations and vaccinations is insignificant.

In the next subsection, we report the results of the non-linear estimations.

### Non-linear specification - penalized spline estimation

The above estimations assume a linear relationship between the response variable and the co-variates. The linearity restriction can be quite restrictive and could amount to model mis-specification if the true relationship was non-linear. Hence in this sub-section, we use a semi-parametric method to estimate the relationship between infections and vaccinations (hospitalizations) without any linearity assumptions. We rely on the estimated degrees of freedom (edf) of the penalized spline estimations to determine the true functional form of the relationships (see Puetz and Kneib (2018), for detailed information on panel splines). Our general model is of the form:

2 $$\begin{aligned} I_{i,t} = \mu _{i} + f(V_{i,t}) + g\left( Z_{i,t}^{T}\right) + \sum _{h=1}^{3}\gamma _{h} D_{h} + \epsilon _{i,t} \end{aligned}$$

where the vector $I_{i,t}$ is the response variable which represents infections in the first specification and hospitalizations in the second specification.

*V* represents the variables that enter the model non-linearly, hence $f(V_{i,t})$ is the penalized function which is orthogonal to the linear part of the model. $Z_{i,t}$ are other control variables and $g\left( Z_{i,t}^{T}\right)$ is the function to be estimated, $\gamma$ measures the impact of the seasons that are modelled linearly, *D* represents the seasonal dummies and $\epsilon _{i,t}$ is the uncorrelated error term assumed to have a zero mean and a constant variance.

Table 3 reports the outcome of the panel spline estimations. The upper part of the table gives the average value of the estimated respective coefficient and the lower part illustrates the degree of non-linearity indicated by the estimated degrees of freedom, where the non-linearity is stronger the higher the value of the edf is and $edf=1$ indicates a linear relationship.

**Table 3:** Estimation results - Panel Spline (Semi-parametric specification)

	*Response variable: Infections*			*Response variable: Hospitalizations*		
*Variables*	1	2	3	4	5	6
*Vaccination*	0.314***	0.133***		0.003	$-$0.093**	
	(0.021)	(0.020)		(0.041)	(0.039)	
*Testing*		0.447***	0.411***		$-$0.022	0.211***
		(0.019)	(0.032)		(0.041)	(0.048)
*Lagged Govt Policy*		$-$0.130***	$-$0.282***		0.285***	$-$0.082
		(0.025)	(0.037)		(0.035)	(0.051)
*Spring*	0.205***	0.92***		0.819***	0.588***	
	(0.063)	(0.062)		(0110)	( 0.099)	
*Summer*	$-$0.424***	$-$0.284***		$-$0.468***	$-$0.275**	
	(0.049)	(0.051)		(0.122)	(0.108)	
*Winter*	0.722***	0.649***	0.239***	1.062***	0.484***	0.711***
	(0.052)	(0.049)	(0.092)	(0.094)	(0.090)	(0.125)
*Booster*			0.471***			$-$0.115*
			(0.037)			(0.069)
*Infections*					0.489***	0.415***
					(0.043)	(0.054)
			Non-Parametric			
*edf(Vaccinations)*	3.09***	6.104***		4.238***	1.001***	
*edf(testing)*		6.696***	6.392**		4.548**	2.063***
*edf(Policy lag)*		5.872***	14.434***		4.522***	3.308**
*edf(Boosters)*			5.903***			
*edf(Infections)*					7.724***	4.776***
*Adj* $R^{2}$	0.27	0.42	0.43	0.37	0.56	0.60
*Observ*	1,888	1,888	625	522	522	200

Standard errors are indicated in parenthesis. *, ** and *** indicates statistical significance at 10%, 5% and 1%, respectively

Model 1 – Only vaccination as a regressor together with seasonal dummies

Model 2 – All regressors

Model 3 – Boosters as a regressor

Model 4 - Only hospitalization as a regressor with seasonal dummies

Model 5 - All regressors

Model 6 - Boosters as a regressor

Table 3 confirms the result with respect to the relation between infections and vaccinations and boosters. It can be seen that the results are identical to those of the panel fixed effects estimations and to those of the GMM estimations, from a qualitative point of view. In particular, vaccinations and boosters are again characterized by a positive relation with the infections on average. As regards the relation between hospitalizations and vaccinations the outcome is again mixed. In model 4 of table 3 the relation is statistically insignificant while it is negative and statistically significant for model 5. When booster is considered as an explanatory variable we get a negative effect that, however, is significant only at the 10% level. Hence, we conclude that there is only weak empirical evidence for the negative effect of vaccinations on hospitalization.

## Conclusion

In this paper we have empirically analyzed the relation that exists between infections with the SARS-CoV-2 virus and the hospitalization rate, respectively, as the dependent variables and the vaccinations against COVID-19 as the independent variable, where we controlled for the effect of other potentially relevant variables. To get a reliable picture of the effects of the true data generating process and to see how robust the results are, we resorted to three different estimation methods: panel fixed effects estimation, GMM estimation and penalized spline estimation.

As regards hospitalizations, six out of ten estimations yielded a statistically insignificant relationship between hospitalizations and vaccinations, three estimation results were weakly statistically significant with a negative coefficient and one indicated a statistically significant negative relation. Hence, our overall conclusion is that the empirical evidence that vaccinations reduce hospitalizations is low. However, it must be recalled that we consider the number of hospitalizations relative to the total population and not relative to the number of infected persons. With respect to the infection rate, our estimations suggest that vaccinations do not reduce the rate of infections, but, on the contrary are positively correlated with the share of vaccinated people. Nine out of ten estimations yielded a statistically significant positive relationship between vaccinations and the rate of infections.

Our analysis should not be misinterpreted as an argument against vaccinations. Vaccinations may be beneficial for persons depending on each person’s individual health characteristics. But, we have found strong empirical evidence that the vaccinations are not associated with positive externalities, implying that society does not benefit from each person being vaccinated since the vaccinations do not prevent infections and transmission of the SARS-CoV-2 virus. This holds because none of our estimations yielded a statistically significant negative relationship between infections and the vaccinations. Hence, our conclusion is that the vaccinations cannot end the pandemic and relying on vaccinations alone is short-sighted. As regards the implications of our analysis with respect to health policy, the development of effective medicines, such as the monoclonale antibody therapy, for example, that offers a good cost-benefit ratio, should be seen as an additional measure. However, that has received little attention due to the focus on the vaccinations (see Richter-Kuhlmann 2021).

## Supplementary Information

Below is the link to the electronic supplementary material.

Supplementary file 1 (pdf 549 KB)

## Data Availability

The dataset used for this study is available upon request from the corresponding author.

## Footnotes

1 All data are taken from Ritchi et al. (2020) that is continuously updated.

2 We do not distinguish between intensive care unit and regular hospitalization.

3 In table 6 in the Appendix we report the estimation result for the fixed effects estimation with non-standardized data that yield the same outcome from a qualitative point of view.

4 The figures 2 - 4 in the Appendix show the actual time series and the fitted ones.

5 As regards hospitalization, observations for 9 European countries are available for the same time intervals as in table 1.
