# Supplementary material for: Effects of the vaccination against SARS-CoV-2 on infections and on hospitalizations in European countries
Source: SN Bus Econ. 2023 Feb 10;3(3):67. doi: 10.1007/s43546-023-00445-0 (PMC9911336; doi:10.1007/s43546-023-00445-0)
Supplement: Supplementary file 1 — (pdf 549 KB) [file 43546_2023_445_MOESM1_ESM.pdf]

6 Appendix

Table 4: Correlation Matrix

	Infections	Vacc	Testing	Boosters	Policy	Hosp
Infections	1					
Vacc	0.318	1				
Testing	0.568	0.335	1			
Boosters	0.654	0.530	0.422	1		
Policy	-0.099	-0.275	0.083	-0.086	1	
Hosp	0.355	-0.018	0.215	0.277	0.133	1

Figure 1: Pairwise scatter plot

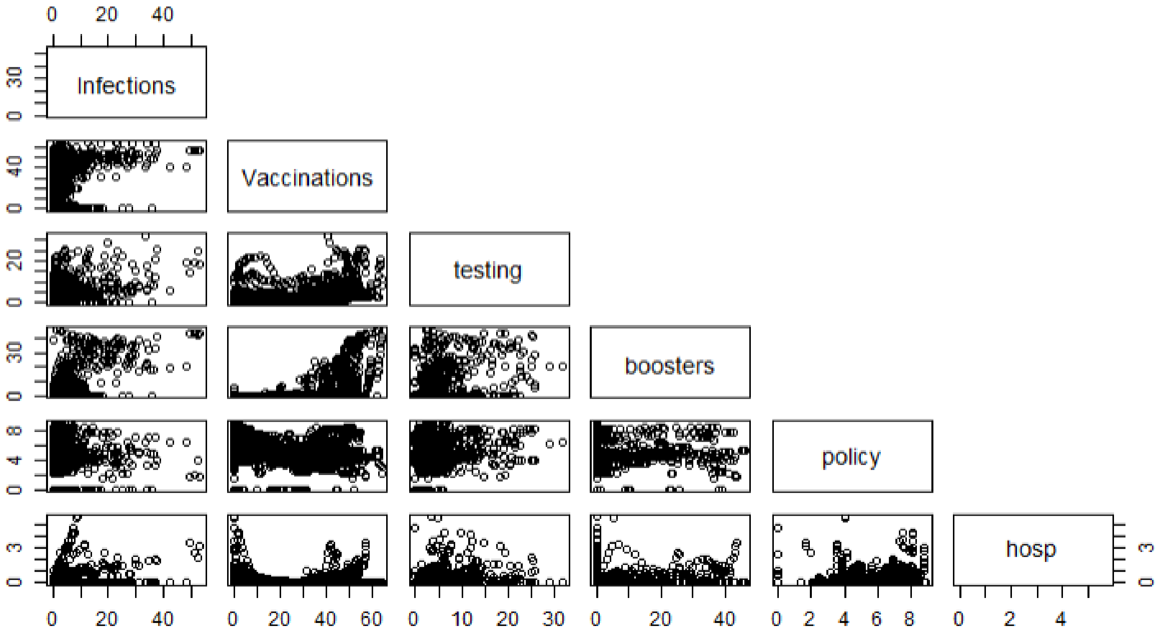

Table 5: Panel Stationarity Test

	IPS- Test		LLC - Test		
Variables	Intercept	Trend	Intercept	Trend	Verdict
Infections	-15.36***	-16.19***	-11.96***	-16.91***	Stationary
Vaccinations	-10.52***	-10.84***	-5.53***	-7.81***	Stationary
Policy	-11.41***	-11.83***	-6.35***	-8.98	Stationary
Testings	-16.78***	-17.708***	-13.63***	-19.27***	Stationary
Hospital admissions	-6.60***	-6.486***	-4.88***	-6.90***	Stationary
Boosters	-12.28***	-12.79***	-8.15***	-11.54***	Stationary

Results of panel stationarity tests, IPS (Im, Pesaran and Shin test) and LLC (Levin Lin and Chu test). The null hypothesis for both tests implies non-stationarity. The table reports the test statistics for both tests. *,**,*** represents rejection of the null hypothesis at 10%, 5% and 1%, respectively.

Table 6: Fixed effects estimation using non-standardized variables

	Response variable: Infections		Response variable: Hospitalizations	
Variables	1	2	3	4
Vaccination	0.067***		-0.002	
	(0.012)		(0.002)	
Testing	0.653***	0.652***	-0.028**	0.028**
	(0.118)	(0.146)	(0.013)	(0.012)
Lagged Govt Policy	-0.832***	-1.144***	0.128***	-0.005
	(0.188)	(0.263)	(0.038)	(0.019)
Spring	2.125***		0.186	
	(0.456)		(0.130)	
Summer	-0.902***		-0.234***	
	(0.279)		(0.085)	
Winter	4.303***	1.520**	0.196	0.378***
	(0.508)	(0.688)	(0.122)	(0.128)
Booster		0.345***		-0.008
		(0.050)		(0.006)
Infections			0.055***	0.040***
			(0.008)	(0.006)
Adj R^2	0.37	0.46	0.52	0.60
Observ	1,920	702	480	208

Newey-West HAC standard errors are indicated in parenthesis. *, ** and *** indicates statistical significance at 10%, 5% and 1%, respectively.

Figure 2: Actual versus fitted values (Infections) based on fixed effects estimation

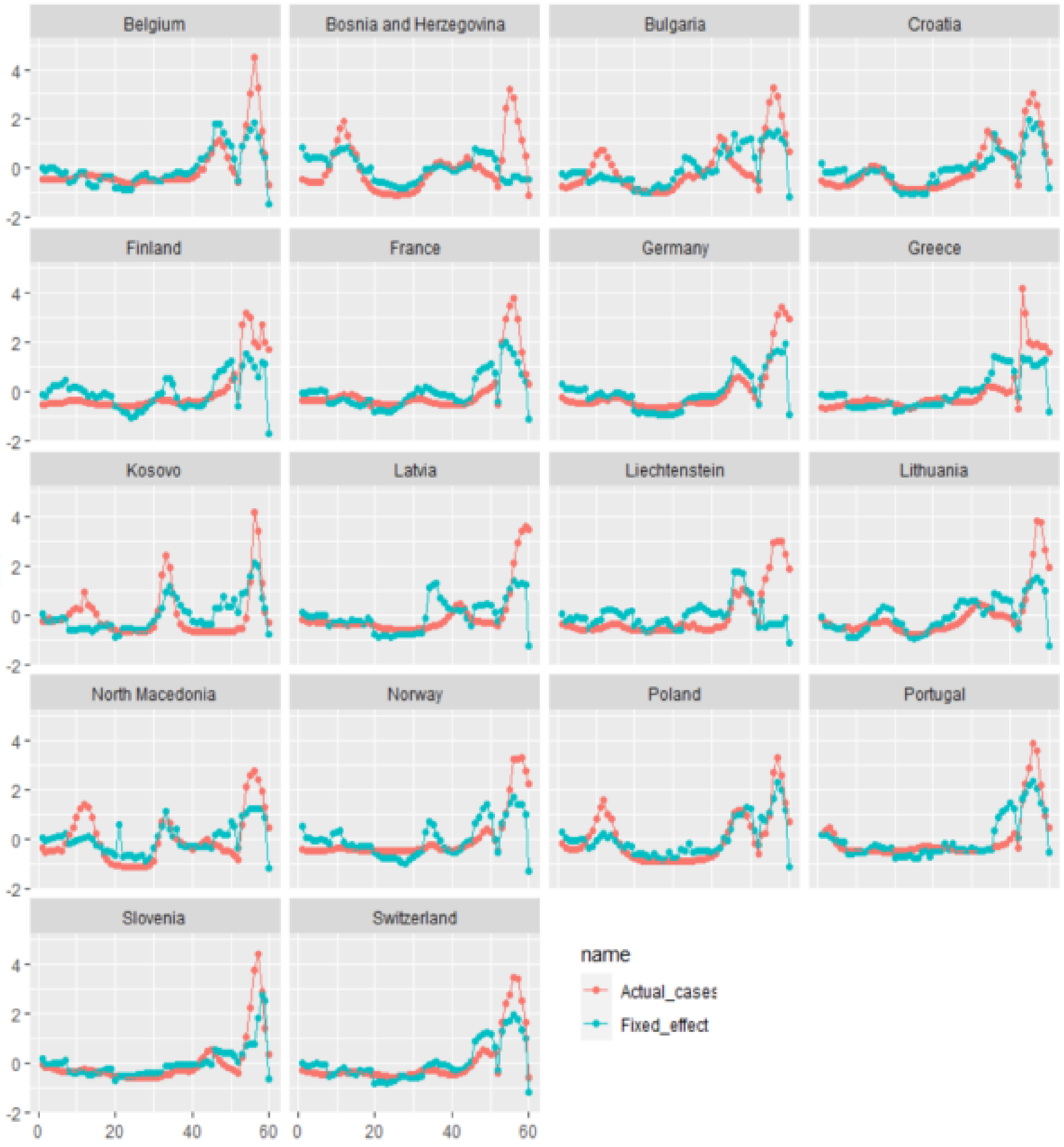

Figure 3: Actual versus fitted values (Infections) based on fixed effects estimation

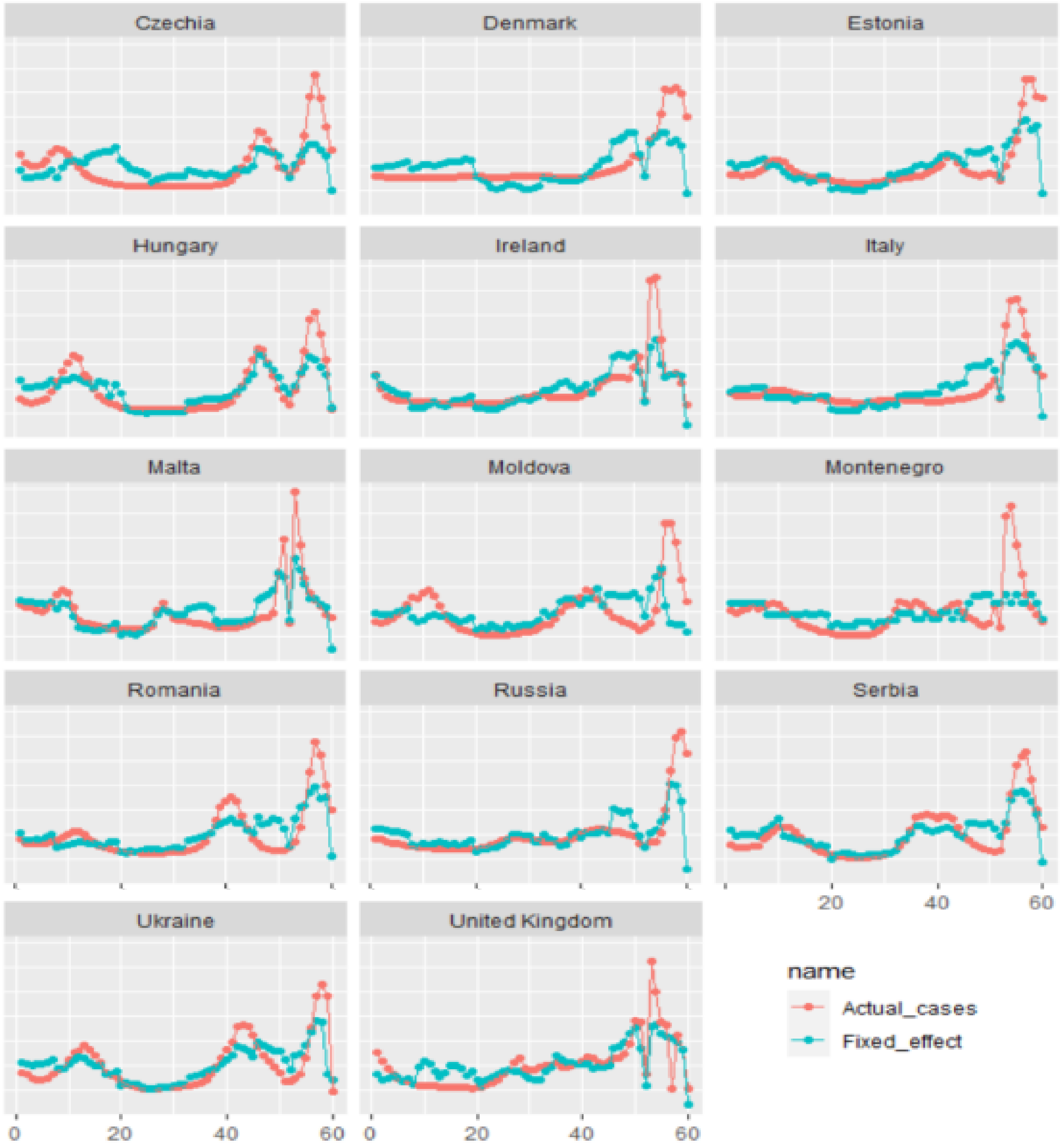

Figure 4: Actual versus fitted values (Hospitalization) based on fixed effects estimation

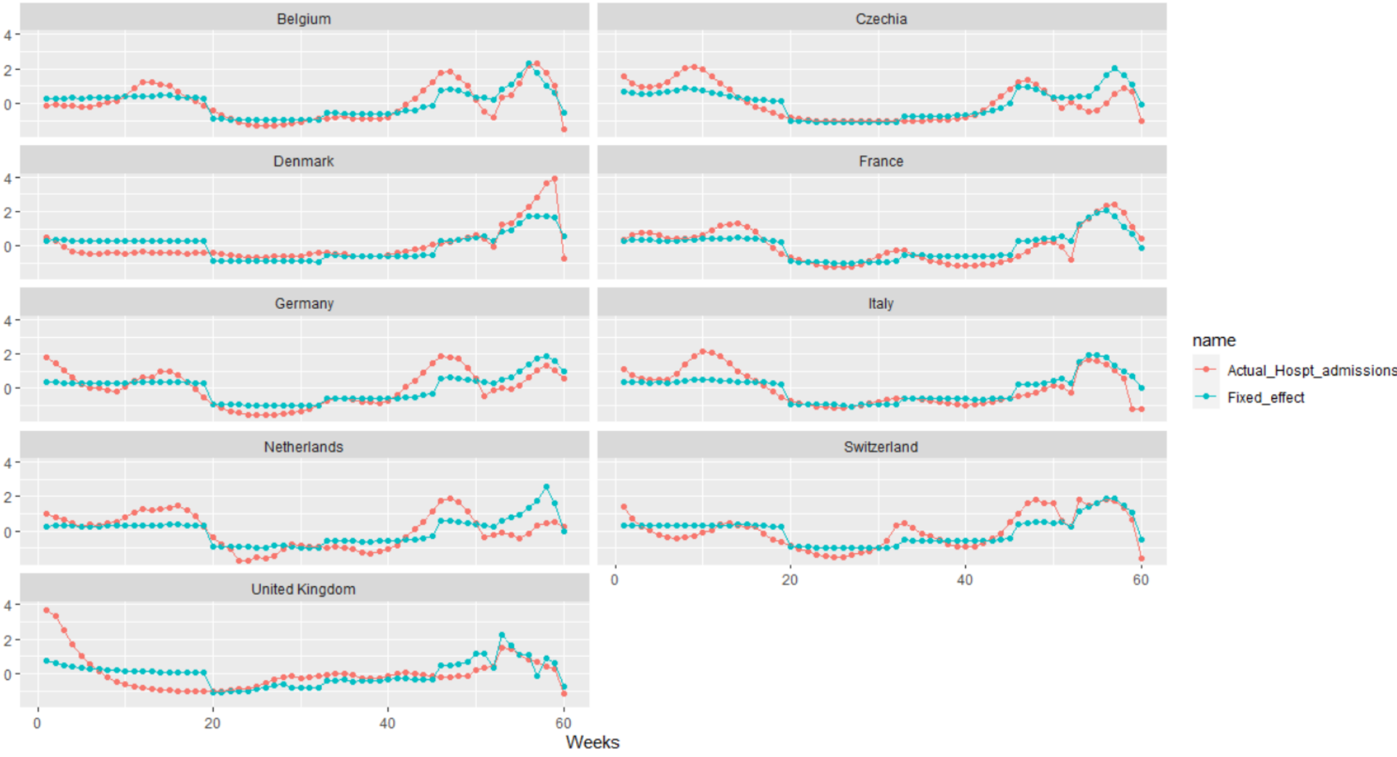

Table 7: Random effect model

	Estimate	Stand error	P-value
Intercept	-0.253***	0.0499	0.000
Vaccinations	0.2501***	0.0256	0.000
Testing	0.3661***	0.0268	0.000
Lagged govt policy	-0.199***	0.0286	0.000
Spring	0.2875***	0.0657	0.000
Summer	-0.1919***	0.0549	0.000
Winter	0.7117***	0.0517	0.000
Adj R^2	0.35		
Obsrv	1920		

Table 8: Hausman Test

	Estimate
Chisq	141.83
Degree of freedom	6
P-value	2.2e-16

Table 9: Multicollinearity test using variance inflation factor

Variable	Vaccination	Testing	Lagged govt policy	Spring	Summer	Winter
VIF	1.335	1.314	1.058	1.766	1.442	1.422
